# Chiropractic students’ perceptions of barriers and facilitators to joining a professional association”

**DOI:** 10.1186/s12998-019-0285-4

**Published:** 2019-11-27

**Authors:** Stanley I. Innes, Norman Stomski, Jean Theroux

**Affiliations:** College of Science, Health, Engineering and Education, Murdoch University, 90, South Street, Murdoch, Western Australia 6150 Australia

**Keywords:** Chiropractic, Professional association, Membership

## Abstract

**Background:**

In Australia, about 1 in 3 chiropractors choose not to belong to either of the two professional associations and this is considerably lower compared to other health professional organisations in this country. The reasons for this remain unknown. We sought to explore possible reasons by asking chiropractic students their perceptions of barriers and facilitators to joining a professional association. However, we were unable to identify validated survey instruments that could be used to obtain information about reasons for joining health professional associations.

**Aim:**

Therefore, the objectives of this study were to: 1) develop a survey instrument that captures information about what influences chiropractic students in joining professional association; and 2) identify factors that promote association membership among chiropractic students.

**Methods:**

A literature review was undertaken to identify known determinants of professional association membership and were used to construct a preliminary survey instrument, which comprised 47 items. Six fourth-year chiropractic students assessed the preliminary survey instrument’s content validity. Principal components analysis was used to establish the structure of the scales. Cronbach’s alpha was derived to determine whether all items in each scale tapped a discrete construct. Logistic regression was used to examine the association between the scale scores and having joined a chiropractic professional association.

**Results:**

In March 2019, 348 chiropractic students from Murdoch University (71.0%) responded to a voluntary, anonymous questionnaire. Principal components analysis resulted in the retention of 21 items that strongly loaded onto 6 factors. Internal consistency was found to be adequate. The results of the logistic regression analysis demonstrated that only “development of the profession” was significantly associated with have joined a professional chiropractic association (*p* = 0.049, OR = 2.22; 95% CI = 1.26–3.40).

**Conclusion:**

Chiropractic organisations can probably most effectively increase membership numbers through raising awareness of their contribution to the development of the profession.

## Introduction

In Australia, about 1 in 3 chiropractors choose not to belong to either of the two professional associations [1, 2]. This phenomenon is not representative of all health professional organisations in Australia. For instance, over 80% of physiotherapists and osteopaths are members of a professional association [3, 4]. This raises the question “Why do approximately one in three Australian chiropractors not join a professional association?”

### Theoretical perspectives about joining professional associations

From a sociological perspective, motives for professional association membership can be interpreted in terms of Weber’s general categories of social action [5]. Here, people join on instrumental-rational motives i.e. organize themselves to assert their personal interests, enhance their personal reputation, and gaining access to desired goods. Also, they join for rational reasons of principle of solidarity or ideological (political, ethical or religious) convictions, as well as to be emotionally associated with the community. Finally, they join because of traditional motives, i.e., parents did.

### Factors linked to chiropractic association membership

Only one study could be found that explored chiropractors’ reasons to join a chiropractic association. That study asked Welsh Institute of Chiropractic Alumni about what influenced their decision to join the United Kingdom Chiropractic Association [6]. A majority of the respondents indicated that the following ten factors were important: promotion of public awareness of chiropractic; access to professional indemnity insurance; professionalism of the association; identity of the association; positive attitude to research; workplace support and advice; access to events, courses, and seminars; continuing professional development activities; cost of membership; and addresses my area of interest.

### Factors linked to non-chiropractic health professionals association membership

Given the dearth of studies examining influences on chiropractic association membership, it would be worthwhile to consider studies of other health professionals to develop a broader understanding of association membership [7–11]. These studies have identified both inhibitors and facilitators to joining a professional association.

In terms of inhibitors, it has been suggested that society as a whole has shifted away from belonging to large groups, since the focus in modern society tends to be on individual rather than community lives [8]. This is reflected by health professionals increasingly placing more importance on balancing family commitments and avoiding activities that may impinge on these commitments [11]. Other inhibitors include perceived lack of personal relevance among younger health professionals [8], cost of annual dues [9], unprecedented levels of student debt [9], and time constraints [10]. Finally, the behaviour of professional associations may also be an inhibitor, as disagreement with association positions and policies [9], and scandals within associations [7], have both resulted in lower levels of membership.

Scant studies have examined facilitators of health professionals’ association membership. That said, it appears that the most important predictor involved the individual’s perception of the value of association membership [12]. In particular, an individual’s decision to join or remain in a professional association depends on whether they perceive that the benefits outweigh the costs [12]. Other factors that may influence health professionals’ decision to belong to professional associations include opportunities for continuing education, access to academic journals, and networking [12, 13]. Overall though, the lack of research in this area suggests that further studies are warranted to better understand health professionals’ motives for joining professional associations.

Promoting association membership during the transition from completing undergraduate training to entering employment may be especially important, as research in the general population has shown that people become increasingly less likely to join a professional organisation the longer they delay after joining the workforce [5, 14]. Consequentially, factors that may inhibit or promote membership in both future non-members and members of professions can be conveniently investigated while still at an undergraduate level.

### Objectives

In light of the aforementioned relatively low rate of Australian chiropractor association membership, and the potentially important transition period from student to practitioner, the purpose of this study was to identify factors that may enhance the likelihood of chiropractic students belonging to such associations. However, we were unable to identify validated survey instruments that could be used to obtain information about reasons for joining health professional associations. Therefore, the objectives of this study were to: 1) develop a survey instrument that captures information about what influences chiropractic students in joining professional association; and 2) identify factors that promote association membership among chiropractic students.

## Method

### Development of the survey instrument

We followed the AMEE guidelines for developing a questionnaire for educational research [15]. A literature review was undertaken to identify known determinants of professional association membership. These determinants were synthesized to construct a preliminary survey instrument, which comprised 47 items (See Additional file 1). Six fourth year Murdoch University chiropractic students then assessed the preliminary survey instrument’s content validity. Each of the six students assessed each item using four categories: not relevant; needs major revision; needs minor revision; and very relevant. Consistent with guideline recommendations, a value of one was awarded to either the “needs minor revision” or “very relevant” categories, and zero was awarded to either the “not relevant” or “needs major revision” categories. An Item- Content Validity Index (I-CVI) was calculated for each item by summing the values for each rater and then dividing by the number of raters. An item was retained if its I-CVI was greater than 0.79 [16, 17]. Of the 47 questionnaire items reviewed, 3 recorded an I-CVI below 0.79 and were subsequently deleted.

Principal components analysis with a varimax rotation was then used to further refine the structure of the questionnaire after data collection had been completed subsequent to its dissemination among all students enrolled in the chiropractic program at Murdoch University. Items with loadings below 0.45, cross-loadings above 0.32, or inter-item correlations below 0.30 were individually deleted, and the component solution was then re-extracted. Internal consistency was examined by calculating Cronbach’s alpha for each factor and the total questionnaire score. The results of the principal components analysis identified six theoretical constructs that underpinned students’ reasons for joining a professional chiropractic association. These constructs, which were grouped into questionnaire scales, comprised: 1) development of the profession; 2) intra-professional communication; 3) practice development; 4) perceived reputation; 5) personal values; and 6) networking. The Cronbach alpha values for these scales ranged from 0.70–0.90, which indicated that all scales had adequate levels of internal consistency.

### Recruitment

Prospective participants comprised all students enrolled in the chiropractic program at Murdoch University, who were informed that participation was voluntary, and no implications would arise if they did not participate. Participants were enrolled through classroom and e-mail invitations. Informed consent was obtained before participants completed an online questionnaire. The questionnaire consisted of the scales detailed in the above section, 15 initiatives that professional associations could potentially use to induce students to take up memberships, and demographic items that captured details about age, gender, year of study, and professional association membership. Anonymity was preserved by ensuring that responses to the survey instrument were not linked to a unique identifier. Ethical approval for this study was obtained from the Murdoch University Human Research Committee (approval number 2019/010).

### Sample size

Given an population size of 490 students, 3% margin of error, and 5% level of acceptable risk, 336 returned questionnaires were required to generalise the findings to the Murdoch University chiropractic student population [18].

### Data analysis

All data was analysed in SPSS v.24 and reported descriptively. Logistic regression was used to examine the association between the scale scores and having joined a chiropractic professional association. Age and gender were entered as confounders into the logistic regression model.

## Results

### Study population

Almost three-quarters of the students enrolled in the chiropractic program at Murdoch University returned completed questionnaires (71.0%; *n* = 348/490). Participants were evenly distributed regarding gender (female = 49.4%). The mean age of the participants was 22.2 years (SD = 4.4). Almost half of the participants were enrolled in year one (27.3%) or year two (23.9%), and the remainder were distributed across years three (17.8%), four (14.4%) and five (12.9%). Finally, half of the participants had already joined a professional association as a student member (49.8%).

### Mean values for the questionnaire scales and association with joining a chiropractic organisation

Table 1 displays the mean values for the six questionnaire scales. The “development of the profession” and “practice development” scales recorded the highest mean values. However, the logistic regression analysis revealed that only “development of the profession” was significantly associated with having joined a professional chiropractic association (*p* = 0.006, OD =2.22; 95% CI = 1.26–3.90) (Table 2). Table 3 displays the mean values for the scales segmented by respondents who have or have not joined a professional chiropractic association.

**Table 1:** Scale mean values

Scale	Mean^a^ (SD)	Scale	Mean^a^ (SD)
Development of the profession	4.3 (3.5)	Perceived reputation	3.1 (2.2)
Intra-professional communication	3.5 (2.5)	Personal values	2.6 (3.5)
Practice development	4.2 (2.1)	Networking	3.7 (2.1)

^a^Note that mean values have been divided by number of items within a scale to facilitate comparisons, but standard deviations remain unaltered

**Table 2:** Results of the logistic regression analysis

	*p* value	OD	95% CI
Development of the Profession	0.006	2.22	1.26–3.90
Personal Values	0.91	1.02	0.76–1.37
Practice Development	0.38	0.83	0.54–1.27
Networking	0.85	1.04	0.71–1.53
Intra-Professional Communication	0.31	1.19	0.85–1.67
Perceived Reputation	0.45	0.91	0.72–1.16
Age	0.07	1.062	1.0.-1.13
Gender	0.09	0.65	0.39–1.07

*OD* Odd Ratio, *CI* Confidence Interval

**Table 3:** Scale mean values segmented by respondents who have joined or not joined an association

Scale	Mean* (SD) Joined Association	Mean* (SD) Not Joined Association	Scale	Mean* (SD) Joined Association	Mean* (SD) Not Joined Association
Development of the profession	4.39 (0.50)	4.13 (0.61)	Perceived reputation	3.57 (0.82)	3.39 (0.82)
Intra-professional communication	3.12 (1.12)	3.12 (1.10)	Personal values	2.52 (0.87)	2.58 (0.88)
Practice development	4.26 (0.69)	4.19 (0.72)	Networking	3.69 (0.73)	3.71 (0.74)

*Note that mean values have been divided by number of items within a scale to facilitate comparisons, but standard deviations remain unaltered

### Frequencies for influence of external factors on joining chiropractic associations

External factors were broadly conceptualised as influences that do not occur within the individual, but instead arise from the environment or other individuals. As such, the scales “development of the profession”, “practice development”, “perceived reputation”, and “intra-professional communication” captured information about external factors. Table 4 displays the item frequencies for the influence of external factors on joining chiropractic associations. Notably, almost all of the items within the “development of the profession” and “practice development” scales were endorsed by more than four in five participants. On the other hand, less than half of the participants endorsed items within the “perceived reputation” scale.

**Table 4:** Frequencies for influence of external factors on joining chiropractic associations

Please indicate below how likely it would be for the following issues to influence your decision to join a chiropractic professional association. E.g., Chiropractic Australia (CA) or the Australian Chiropractic Association (ACA).	Highly Likely/Likely
Development of the Profession	
Organisation that promotes public awareness of chiropractic	87.3
Overall professionalism of the association	86.7
Promotes research	82.7
Workplace support and advice such as locum or associate contracts	76.2
Access to Continuing Professional Development (CPD) activities	82.0
Lobbying government and Medicare for more inclusion of chiropractic	88.1
Intra-Professional Communication	
Influence of other chiropractors and students I know	62.0
Professional association newsletter	51.3
Social media presence	40.2
Perceived Reputation	
The professional associations have poor public reputations	42.7
Professional associations don’t represent my own personal chiropractic values	35.1
Practice Development	
Professional associations keep me up to date with best practice	82.2
Professional associations keep me up to date with new information about my profession	86.1
Professional associations offer me a way to improve my standards of practice	87.0

### Frequencies for influence of internal factors on joining chiropractic associations

Internal factors relate to the association between individual’s behaviour and their abilities, traits, or feelings. These dispositional attributions were reflected by the items within the “personal values” and “networking” scales. Table 5 displays the item frequencies for the influence of internal factors on joining chiropractic associations. “Personal values” appeared to be of relatively little importance as only around one in five participants stated that it influenced their decision to join professional associations. “Networking” though, seems important as about three-quarters noted that two of the three items were likely to affect their decisions.

**Table 5:** Frequencies for Influence of Internal Factors on Joining Chiropractic Associations

How likely would it be for the following personal views to influence your decision to join a professional association	Highly Likely/Likely
Personal Values	
I don’t believe it’s important to belong to a professional association	16.8
I don’t agree with either of the professional association positions and policies	21.3
I can get everything the associations offer without belonging to one	19.6
I’ve never seen the value of belonging to a professional association	15.8
Networking	
Belonging to a professional association supports the professions development	52.5
I would join if I knew the educators, I respect were professional association members	71.8
Professional associations allow me a way to keep me involved in chiropractic	74.3

### Frequencies for influence of initiatives on joining chiropractic associations

Table 6 displays the item frequencies for the influence of initiatives on joining chiropractic associations. As can be seen from the Table, half of the initiatives were endorsed by at least four in five participants.

**Table 6:** Frequencies for influence of initiatives on joining chiropractic associations

How likely are the following initiatives to influence you to become a member of a professional association?	Highly Likely/Likely
A yearly email invitation to join	45.3
Alternative payment methods, such as being able to direct debit once a month	55.9
Congratulatory email on graduation	54.7
Annual competitions relevant to chiropractic	50.7
Scholarships	85.3
Research grants	82.2
Fees for recent grads are either reduced or waived	85.2
Website regularly conveys relevant information	71.5
Website videos of what they have achieved	58.1
Member benefits	89.6
Offer post graduate programs such as paediatric care, women’s health & aged care	84.0
A personal printed invitation to join a professional association through the student teaching clinic	56.0
The more support given by the professional association the more likely I am to join	81.5
Free “stuff”	67.7
A clearly described path for the future	79.8

## Discussion

We developed a valid instrument that capture details about factors that influence chiropractic students in joining associations. This instrument comprised six discrete constructs, which were: development of the profession; intra-professional communication; practice development; perceived reputation; personal values; and networking. Of these constructs, it appears that chiropractic associations would most effectively increase membership numbers if they focused on issues associated with “development of the profession”. The construct that we conceptualised as “development of the profession” consisted of six discrete elements (see Table 4). Each of these elements was highly endorsed, which suggests that communication strategies designed to increase member numbers could focus on any of the elements.

Our findings were consistent with the results of studies in other health professions, in that opportunities for ongoing education and networking were considered to be important factors in influencing association membership [12, 13]. Moreover, the findings of this study were largely in agreement with the only other chiropractic study that examined factors involved with joining a professional chiropractic association [6]. In particular, “promoting public awareness of chiropractic”, “promoting research”, and “workplace support” were highly endorsed by respondents in both chiropractic studies as important in influencing the decision to join a professional association [6].

In comparison to external factors, personal factors were perceived as less important in influencing decisions about joining chiropractic professional associations. This was an interesting finding as personal attitudes can be difficult to shift and resources may be better spent on other strategies that alter external factors [19, 20].

Finally, in terms of initiatives that were likely to influence decisions about joining a professional chiropractic association, we identified seven strategies that were endorsed by least four in five participants. Decisions about which of these strategies an organisation may implement could be guided by pragmatic considerations of each strategy’s ease of dissemination and cost effectiveness.

### Limitations

This study was undertaken at a single Australian university. Whether the views of the respondents in this study are representative of the perceptions of chiropractic students at other Australian universities is unclear. Nonetheless, the number of completed questionnaires exceeded the required number, and the demographic characteristics of the respondents were representative of the overall student cohort, which lends tentative support for the generalisability of our findings. In addition, further psychometric testing of the instrument constructed for this study would be beneficial. We developed an instrument that has established content validity and adequate levels of internal consistency, but it would be worthwhile to undertake additional studies that examine the instrument’s intra-rater reliability, construct validity and responsiveness. Moreover, using the same sample to validate the questionnaire and assess chiropractic students’ views about association membership may have resulted in overfitted data [21]. Additional studies should be conducted in other chiropractic student populations to consolidate our findings regarding predictors of professional chiropractic association membership.

There are two chiropractic associations in Australia that chiropractic students could potentially join. We did not ask which association students had chosen to join, nor if they planned to join either of these after graduation. Exploring such differences may further inform our understanding of factors influencing the likelihood of future professional chiropractic association membership.

Student membership is free and the reality of the impost of regular financial dues after graduation may change these findings. A longitudinal study following these students would reveal if cost is an important factor. Another potential investigation could be undertaking qualitative interviews with longstanding non-member clinicians to identify their reasons for not joining professional chiropractic associations.

## Conclusion

Our findings demonstrated that Australian chiropractic organisations can probably most effectively increase membership numbers through raising awareness of their contribution to the development of the profession. Alternatively, endeavouring to change dispositional attributions, particularly personal views about professional associations, appears to be the least effective approach to increase association membership.

Finally, in terms of initiatives that were likely to influence decisions about joining a professional chiropractic association, we identified seven strategies that were endorsed by least four of five participants. Decisions about which of these strategies an organisation may implement could be guided pragmatic considerations of each strategy’s ease of dissemination and value for money.

## Supplementary information

**Additional file 1.** Original 47-item questionnaire.

## Data Availability

Not applicable.
