# Supplementary material for: Chiropractic students’ perceptions of barriers and facilitators to joining a professional association”
Source: Chiropr Man Therap. 2019 Nov 27;27:67. doi: 10.1186/s12998-019-0285-4 (PMC6880539; doi:10.1186/s12998-019-0285-4)
Supplement: Supplementary file 1 — Additional file 1. Original 47-item questionnaire. [file 12998_2019_285_MOESM1_ESM.docx]

Additional file 1

| **Please indicate below how likely it would be for the following issues to influence your decision to join a chiropractic professional association. E.g., Chiropractic Australia (CA) or the Australian Chiropractic Association (ACA).** | **Highly Unlikely** | **Unlikely** | **Neutral, neither likely or unlikely** | **Likely** | **Highly Likely** |
| --- | --- | --- | --- | --- | --- |
| 1. Organisation that promotes public awareness of chiropractic |  |  |  |  |  |
| 1. Provides access to professional insurance at a reduced cost |  |  |  |  |  |
| 1. Overall professionalism of the association |  |  |  |  |  |
| 1. Promotes research |  |  |  |  |  |
| 1. Workplace support and advice such as locum or associate contracts |  |  |  |  |  |
| 1. Access to events \ courses \ seminars |  |  |  |  |  |
| 1. Access to Continuing Professional Development (CPD) activities |  |  |  |  |  |
| 1. Affordability of membership |  |  |  |  |  |
| 1. Lobbying government and Medicare for more inclusion of chiropractic |  |  |  |  |  |
| 1. Lobbying AHPRA and the Registration Board for advancement of the profession |  |  |  |  |  |
| 1. Lobbying private insurance agents (E.g. Bupa) for better funding of chiropractic |  |  |  |  |  |
| 1. Influence of other chiropractors and students I know |  |  |  |  |  |
| 1. Professional association newsletter |  |  |  |  |  |
| 1. Social media presence |  |  |  |  |  |
| 1. The professional associations have poor public reputations |  |  |  |  |  |
| 1. Professional associations keep me up to date with best practice |  |  |  |  |  |
| 1. Professional associations keep me up to date with new information about my profession |  |  |  |  |  |
| 1. Professional associations offer me a way to improve my standards of practice |  |  |  |  |  |
| 1. A professional association allows ways for me to feel more part of the chiropractic profession |  |  |  |  |  |
| 1. Professional associations don’t represent my own personal chiropractic values |  |  |  |  |  |
| **How likely would it be for the following personal views to influence your decision to join a professional association** | **Highly Unlikely** | **Unlikely** | **Neutral, neither likely or unlikely** | **Likely** | **Highly Likely** |
| 1. I don’t believe it’s important to belong to a professional association |  |  |  |  |  |
| 1. I don’t agree with either of the professional association positions and policies |  |  |  |  |  |
| 1. I can get everything the associations offer without belonging to one |  |  |  |  |  |
| 1. I’ve never seen the value of belonging to a professional association |  |  |  |  |  |
| 1. I want to belong to a like-minded group |  |  |  |  |  |
| 1. By belonging to a professional association I can financially support the development of the profession |  |  |  |  |  |
| 1. I would join if I knew the educators I respect were professional association members |  |  |  |  |  |
| 1. Professional associations allow me a way to keep me involved in chiropractic |  |  |  |  |  |
| 1. I think belonging to a professional association enhances my standing in the community |  |  |  |  |  |
| 1. Please list any other issues you think may be influential in determining whether or not a student may join a professional association. Other . . . . . . . . . |  |  |  |  |  |
| **How likely are the following initiatives to influence you to become a member of a professional association?** | **Highly Unlikely** | **Unlikely** | **Neutral, neither likely or unlikely** | **Likely** | **Highly Likely** |
| 1. A yearly email invitation to join |  |  |  |  |  |
| 1. Alternative payment methods, such as being able to direct debit once a month |  |  |  |  |  |
| 1. Congratulatory email on graduation |  |  |  |  |  |
| 1. Annual competitions relevant to chiropractic |  |  |  |  |  |
| 1. Scholarships |  |  |  |  |  |
| 1. Research grants |  |  |  |  |  |
| 1. Fees for recent grads are either reduced or waived |  |  |  |  |  |
| 1. Website regularly conveys relevant information |  |  |  |  |  |
| 1. Website videos of what they have achieved |  |  |  |  |  |
| 10. Member benefits |  |  |  |  |  |
| 11. Offer post graduate programs such as paediatric care, women’s health & aged care |  |  |  |  |  |
| 12. A personal printed invitation to join a professional association through the student teaching clinic |  |  |  |  |  |
| 13. The more support given by the professional association the more likely I am to join |  |  |  |  |  |
| 14. Free “stuff” |  |  |  |  |  |
| 15. A clearly described path for the future |  |  |  |  |  |
| 16. Please list below any other initiatives you think may influence chiropractic students to join a professional association. Other . . .. . . .. . . . . . .. . . . |  |  |  |  |  |
